# Liver Transplant Imaging prior to and during the COVID-19 Pandemic

**DOI:** 10.1155/2022/7768383

**Published:** 2022-01-12

**Authors:** Maria Irene Bellini, Daniele Fresilli, Augusto Lauro, Gianluca Mennini, Massimo Rossi, Carlo Catalano, Vito D'Andrea, Vito Cantisani

**Affiliations:** ^1^Department of Surgical Sciences, Sapienza University of Rome, Rome, Italy; ^2^Department of Radiological, Oncological, Anatomo-Pathological Sciences, Sapienza University of Rome, Rome, Italy; ^3^Department of Hepato-Bilopancreatic and Transplant Surgery, Sapienza University of Rome, Rome, Italy

## Abstract

**Background:**

The suspension of the surgical activity, the burden of the infection in immunosuppressed patients, and the comorbidities underlying end-stage organ disease have impacted transplant programs significantly, even life-saving procedures, such as liver transplantation.

**Methods:**

A review of the literature was conducted to explore the challenges faced by transplant programs and the adopted strategies to overcome them, with a focus on indications for imaging in liver transplant candidates.

**Results:**

Liver transplantation relies on an appropriate imaging method for its success. During the Coronavirus Disease 2019 (COVID-19) pandemic, chest CT showed an additional value to detect early signs of SARS-CoV-2 infection and other screening modalities are less accurate than radiology.

**Conclusion:**

There is an emerging recognition of the chest CT value to recommend its use and help COVID-19 detection in patients. This examination appears highly sensitive for liver transplant candidates and recipients, who otherwise would have not undergone it, particularly when asymptomatic.

## 1. Introduction

The Coronavirus Disease 2019 (COVID-19) caused by the Severe Acute Respiratory Syndrome Coronavirus-type 2 (SARS-CoV-2) led to unprecedented challenges to healthcare. It is mainly a respiratory disease, with a mild course in most of the cases, but with potentially fatal outcomes for certain population subgroups. Previous reports demonstrated higher risk in immunosuppressed transplant patients, individuals with underlying comorbidities, such as diabetes, age > 70 years, obesity, and cardiac and/or end-stage organ disease [1]. COVID-19 diagnosis is based on the positivity of the reverse transcriptase-polymerase chain reaction (RT-PCR) [2].

Extreme measures, such as the lockdown or red zone instauration, have been the only effective way to counteract the overwhelming pressure by this novel virus. The aim of this review is to provide a comprehensive assessment of the evolution of the diagnostic imaging caused by the pandemic in relation to liver transplantation (LT). We report the standard of practice running until the prepandemic area, followed by the impact of COVID-19 in the use of radiological means to select, manage, and follow up LT patients and donors during these challenging times.

## 2. Liver Transplant Patient Imaging: Guidelines in the Pre-COVID-19 Era

It is crucial to assess the transplant candidate timely by a multidisciplinary team, where the radiologist plays a key role in the early identification of anatomical conditions and disease before they could become symptomatic. The latest European guidelines on the management of the organ donor and the recipient [3] consider the following:

- To acquire a precise evaluation of vascular anatomy
- To acquire a precise evaluation of biliohepatic anatomy
- To evaluate liver parenchymal status and dimensions
- To reveal hidden cancer
- To exclude cholangiocarcinoma in primary sclerosing cholangitis patients
- To evaluate posttransplantation complications

Pretransplant assessment is generally acquired by computed tomography (CT) imaging with intravenous contrast medium administration [3], but multiparametric ultrasound could provide a great contribution to recipient evaluation before and after surgery due to its noninvasiveness, widespread availability, high repeatability, and cost-effectiveness, alongside the option of a bedside execution.

### 2.1. Pretransplant Liver Imaging

An optimal multidisciplinary postoperative management by means of imaging modalities is recommended, with the three-phase intravenous contrast CT scan being the most adopted imaging method [3]. Additionally, transthoracic echocardiography in all LT candidates is valuable.

History of a treated cancer is not an absolute contraindication to LT. One of the eligibility criteria in cancer patients is the recurrence risk of less than 10% [3]. Another criterion is a free-of-recurrence interval time of 5 years, although it may vary considerably according to the type of malignancy.

Hepatocarcinoma (HCC) represents one of the main indications for transplantation by treating both the tumour and the underlying liver disease [4]. Not all HCCs can benefit from transplant treatment, but the effectiveness varies according to the stage of the disease, i.e., tumour stage, liver function, and functional status of the patient.

Both dynamic CT and dynamic magnetic resonance imaging (MRI) with contrast are the best imaging modalities to make HCC diagnosis, although they can underestimate or overestimate the extent in up to 25% of cases, compared with pathological findings of the explanted liver [5]. Extrahepatic staging should include chest CT and abdomen-pelvis CT or MRI.

While on the waiting list, periodic monitoring should be performed by imaging (dynamic CT, dynamic MRI, or contrast-enhanced ultrasound) and *α*-fetoprotein measurements [4].

### 2.2. Posttransplant Liver Imaging

As a complex surgical procedure, accurate imaging is essential to detect early complications; therefore, regular diagnostic follow-up is recommended to improve graft and patient survival. LT complications could be classified into vascular (affecting the hepatic artery, portal vein, and/or hepatic vein) or biliary (involving the biliary tree). There is also a significant number of complications in relation to immunosuppression, more in details on infection, rejection, or malignancy [6].

While preoperative evaluation primarily involves CT and MRI, postoperative management relies on screening with US, eventually followed by deeper definition via additional modalities [7].

Within the first day postsurgery, US is highly recommended for baseline assessment [8, 9], representing the preferred investigation for an initial evaluation of symptomatic patients, especially in the case of young paediatric recipients, in order to obtain an early diagnosis of the postsurgical complication and carry out timely intervention [10].

Generally, in the postoperative period, a colour Doppler study is performed within 24 hours after an uncomplicated transplant, and another is required before discharge, but protocols may vary according to the different centres' practice.

A checklist of the findings to be evaluated could be followed during US examination to improve detection and to discern among the possible pathological conditions, as shown in Table 1.

However, further evaluation may be necessary in case of doubt or inconclusive US examination (CT, MRI, or biopsy). Baheti et al. proposed a diagnostic algorithm for evaluating LT patients with suspected complications [6]. Also, Kimura et al. proposed a diagnostic algorithm to rapidly discover any vascular or biliary complications [9].

Oncological monitoring is relevant in LT patients; in fact, chronic immunosuppression could lead to the development of neoplastic diseases, such as posttransplant lymphoproliferative disorder (PTLD), Kaposi's sarcoma, and nonmelanocytic skin cancers. Observational studies have shown a 2–3-fold increased risk of solid organ cancers and a >30-fold increase in the rate of lymphoproliferative malignancies, compared to the general population [3].

PTLD related to Epstein-Barr virus infection consists of lymphoproliferative cancer, mainly occurring within the first 2 years of follow-up. In this case, abdominal involvement is more common than the extra-abdominal location, with singular or multiple lymphadenopathy manifestation and with or without splenomegaly. The most affected organs with extranodal involvement are the bowel, liver, kidneys, and adrenals, although it has been described as potentially occurring in any part of the human body [11, 12].

In transplant patients with hepatocellular carcinoma (HCC), recurrent HCC may also occur, usually involving the lung and the liver graft, with a median time to relapse of approximately 1 year [13].

Cancer screening must always be performed via endoscopy (colonoscopy, upper gastrointestinal endoscopy, and nasofibroscopy) and/or imaging (CT colonography, chest HRTC, and mammogram) tools, taking into consideration the patient's age, sex, alcohol consumption, and smoking habits.

For follow-up surveillance in patients with previous HCC, an international consensus report from 2012 recommends total-body CT or MRI every 6 to 12 months [4, 7].

Although there have been recent and significant improvements in US, even with contrast medium introduction (CEUS), US is less used than CT or MRI due to the inability to reliably acquire images of the entire liver during a particular contrast phase. However, there are many studies that demonstrate the usefulness of CEUS in the clinical and radiological management of the transplant patient, for example, in the kidney transplant scenario [14].

## 3. New Ultrasound Techniques in Liver Transplantation

### 3.1. Contrast-Enhanced Ultrasound

Colour Doppler US provides a reliable method for screening and evaluation of postliver transplant vasculature, but it has limitations in the assessment of small-calibre vessels. CEUS may overcome it, reducing the number of false-positive cases and thus unnecessary additional investigations, such as CT or angiography; in fact, by using microbubbles as blood pool contrast agents, CEUS allows qualitative and quantitative high-quality real-time evaluation.

CEUS can be used as a problem-solving tool when previous US, CT, and/or MRI imaging report undefined vascular and liver parenchymal findings [15]. In this regard, it could be very useful to visualize the hepatic artery, not otherwise recognizable in B-mode, for example, in conditions occurring with the overlapping of the portal vein or if of small calibre [16].

Another important benefit offered by CEUS is the detection of filling defects, if present, or lumen stenosis. As reported in the European Federation for Ultrasound in Medicine and Biology guidelines [17], CEUS is useful to identify portal thrombus hypervascularization which discriminates HCC portal invasion from bland portal vein thrombosis [18, 19].

Hepatic vein complications are rare (1%) [20] and often difficult to be detected in US (such as in the case of hepatic vein stenosis); when the suspect arises, this requires confirmation via further imaging, generally in the form of CT or magnetic resonance imaging. CEUS might represent instead an exam demonstrating hepatic vein thrombosis or the site of anastomotic narrowing, without the need for further confirmation [16].

Concerning biliary complications, it has been previously reported that the administration of contrast media via the intravenous route is not of sufficient effectiveness, but its usefulness in demonstrating eventually biliary leakage and strictures increases when it is injected into the biliary system through a T-tube (CEUS cholangiography) [21, 22]. Finally, CEUS can also differentiate an infarcted area from an HCC lesion or abscess in the parenchyma.

For the abovementioned reasons, Como et al. suggest an extensive LT patient CEUS-integrated diagnostic workflow [15]. However, there are only few studies in the literature about CEUS applications in the LT setting, making it difficult to ascertain its own role or in combination with other traditional imaging methods, thus limiting significantly its use in the posttransplant setting [16, 23–27].

### 3.2. US Elastography

US elastography examines the intrinsic liver parenchymal state by measuring liver stiffness (LS) that, if high, is considered a marker of liver fibrosis or cirrhosis [28–30].

In the community, elastosonography is emerging as a noninvasive tool to evaluate the fibrosis of the hepatic graft, mainly in the paediatric population, allowing a decrease in the number of invasive procedures, such as liver biopsy.

US-based elastography comprehends different techniques [28, 29, 31], with yet not an established specific role in LT patients. Current available data mainly report on the use of vibration-controlled transient elastography (TE) and point shear wave elastography (pSWE), especially regarding graft rejection.

The controlled attenuation parameter (CAP) is a novel ultrasound-based elastography method for detection of the grade of steatosis, an important parameter in the assessment of a liver donor, and mainly associated with male sex and high body mass index [32].

CAP measures ultrasound attenuation at the central frequency of the VCTE via the M and/or regular probe [33, 34]; its accuracy may be yet affected by variations in cut-off values of steatosis severity or integration with other parameters [35]; therefore, more recently and in other clinical settings, new quantification methods have been proposed. A recent meta-analysis looking at the TE and CAP role in steatosis and fibrosis diagnosis and staging in alcoholic and nonalcoholic liver diseases showed a pooled sensitivity and specificity of CAP of 0.84, 0.83, and 0.78 and 0.83, 0.71, and 0.62 for steatosis grades ≥S1, ≥S2, and =S3, respectively [36].

## 4. Living Donor Imaging

The living donor LT is less common in Western countries, in consideration of the potential risks for the donor and the higher posttransplant complication rate, in comparison to the deceased donation that is instead prevalent. It is yet very important and timely to emphasize the role of living donation to expand the donor pool and allow more transplants, especially in the postpandemic scenario, when due to current theatre restrictions, a growing organ demand is envisaged [37]. During COVID-19, in fact, it has been considered even a more essential resource for leading centres to contrast the mortality increase in candidates awaiting LT [38]. In the current global scenario, it is paramount to achieve an adequate imaging assessment, preferentially to be all performed in a single instance, avoiding in this way unnecessary donor exposure to risks related to hospital admission.

Generally, in standard practice, US is the first-line diagnostic tool to assess potential candidates and exclude focal hepatic lesions. It also assesses the amount of steatosis and provides preliminary vascular anatomy.

However, further assessment with cross-sectional imaging modalities is usually necessary.

CT findings determine split liver volumes, eventual vascular and biliary anatomy variations, and fatty infiltration [39–41]. In more detail, donor liver assessment includes [42] the following:

- Parenchymal evaluation for diffuse liver disease (fatty infiltration) and focal lesion detection
- Liver volume estimation
- Liver vascularization (arterial, portal, and hepatic venous) and variants
- Biliary anatomy and variants

## 5. Liver Transplantation in COVID-19: What the Pandemic Implied for Transplant Programs

The suspension of the surgical activity, the burden of the infection in immunosuppressed patients, and the comorbidities underlying end-stage organ disease have impacted significantly the run of transplant programs. Uncertainty regarding SARS-CoV-2 transmission from the donor to the recipient and the risk of developing COVID-19 posttransplant from sources not related to the donor, as well as facility, logistical, and organizational issues, along with the implications of social distancing for the follow-up, represented the main issues to safely maintain an efficient transplant program [43]. Furthermore, the peak of COVID-19 hospitalization and admissions to intensive care units (ICUs) not only impacted the possibility to treat with dedicated pathways (buildings and staff) non-COVID-19 patients, such as transplant patients, but also importantly affected the overall number of deceased donors in ICU to be considered for the start of the whole transplantation process.

In parallel with the pandemic phases, we could identify three different approaches that have been adopted to carry on the necessary surgical activity [44]:

1. Significant flattening of the observed pandemic curve allows a partial reopening. This includes a stable number of new cases and some stabilization in ward/ICU bed utilization (orange zone)
2. The number of new COVID cases is flat or decreasing for a reasonable period, and ICU and ward bed utilization has been at a stable level at the transplant hospital (yellow zone)
3. Prolonged stability and/or a decrease in COVID activity is observed along with prolonged stability or a decrease in hospital ICU/ward bed utilization. ICUs are running consistently below capacity in the transplant hospital (white zone)

In phases 1 and 2, liver transplant programs have limited living donor activity to moderate to severe sick recipients only and performed deceased donor activity only from standard donors or donors after cardiac death, with a higher chance of primary graft function (i.e., <50 years age). This occurred particularly at the beginning of the pandemic. Only in phase 3, living donor activity slowly came back to normal, with priority given to sicker recipients, while deceased donor activity was run under no restrictions. The distinction between living donation and deceased donation assumes that the former is at too high risk, since the prospective candidate is a healthy individual who does not need to come to the hospital, if not for the wish to donate. It is no surprise therefore that the elective phases (orange, yellow, and white zones) following the lockdown (red zone) impacted more substantially the kidney [45], a non-life-saving organ, in comparison to the liver. In more detail, in Italy, one of the most heavily affected countries by the pandemic, kidney waitlisted candidates dropped to 15% less in comparison to the constant number registered in the last 5 years; this effect was not instead observed for liver transplantation, with the numbers remaining stable throughout the pandemic, demonstrating the necessity to perform this life-saving procedure [46].

Indeed, it is paramount to correctly diagnose COVID-19 disease among transplant patients to prevent and manage this disease, with detrimental outcomes already described in the high-risk transplant subgroup.

It is known that underlying comorbidities increase the overall mortality risk in COVID-19 infection; therefore, patients with chronic liver disease, such as cirrhosis or autoimmune hepatitis, are more vulnerable. Yet, clinical risk factors specific for pathologic hepatic conditions and cancers and liver graft recipients are not clearly defined [47]. Mortality attributable to COVID-19 appears higher in patients with more advanced liver disease [47], and there is no reassuring data in terms of early immunization against the virus in transplanted patients [48]. While data is scarce, the incidence of early posttransplant Coronavirus Disease 2019 (COVID-19) may reach up to 38% [49]; this is acquired generally in the community or during hospitalization. Thus, it was recommended that all patients should be evaluated for COVID-19 routinely and particularly preoperatively and that isolation should be considered for all transplant patients [50], with significant challenges to adequately follow them up.

### 5.1. Recipient and Donor SARS-CoV-2 Screening

A systematic SARS-CoV-2 screening strategy could be implemented for all recipients and donors including the following:

- A questionnaire on prehospitalization symptoms and a clinical examination at hospital admission
- A nasopharyngeal swab for SARS-CoV-2 by RT-PCR IP2/4
- A chest CT scan prior to LT [51]

If all these three screening tests are negative, the LT could be performed.

Unfortunately, testing protocols are not standardized nationally, and current tests have significant false-negative results. Chest CT has a high sensitivity but is not always readily available, and society guidelines do not recommend its use as a routine screening strategy [52].

### 5.2. Preoperative Imaging in Liver Transplantation during the COVID-19 Era

#### 5.2.1. Chest CT

Chest CT is a highly sensitive technique in identifying possible COVID-19 pulmonary involvement [53]. It is a widely used, fast-performing method that should be performed without contrast medium, unless pulmonary embolism is suspected. Its main limitations are the use of ionizing radiation and relatively high costs.

Many studies have shown that CT is more sensitive than the swab for the detection of SARS-CoV-2 infection, although it is not specific, since the same “ground glass” findings may be related to other interstitiopathies [54]. In this regard, a large Chinese analysis on 1014 patients reported lower sensitivity for RT-PCR, with a mean interval time between the initial negative result and the positive result of 5.1 ± 1.5 days, in contrast to the immediate positivity of the chest CT [55].

The European Society of Radiology (ESR) and the European Society of Thoracic Imaging (ESTI) published a document about SARS-CoV-2 pneumonia CT imaging focusing on typical findings in the early COVID-19 diagnosis [56].

According to ESR and ESTI guidelines [56], the most common COVID-19 pulmonary findings in unenhanced thin-section chest CT are as follows:

- Bilateral ground glass opacities, with a predominant peripheral, subpleural location (in the early phase), extensively located into the parenchyma or more focal, with a rounded shape
- Intralobular reticulations superimposed on the ground glass opacities, resulting in a crazy-paving pattern (usually associated with a more severe stage of the disease)

Mucoid impactions, centrilobular nodules, lobar consolidation, lymphadenopathy, or significant pleural effusions are infrequently seen in COVID-19 patients.

The less common chest CT findings reported in 10%–70% of RT-PCR test-proven COVID-19 cases [57] are as follows: consolidation, linear opacity, septal thickening and/or reticulation, air bronchogram, pleural thickening, halo sign/reversed halo sign bronchiectasis, nodules, and bronchial wall thickening.

The distribution of these findings can be unilateral (15.0%), multifocal (63.2%), diffuse (26.4%), and single and/or focal (10.5%), with a peripheral location only (59.0%) or with a central and peripheral location concomitantly (36.2%) [57]. The middle or upper lobe locations are more frequent.

The uncommon chest CT findings in RT-PCR test-proven COVID-19 cases [57, 58] are as follows: pleural effusion, lymphadenopathy, tree-in-bud sign, central lesion distribution, pericardial effusion, and cavitating lung lesions.

Although the CT findings of COVID-19 are characteristic, the final diagnosis requires a positive RT-PCR test.

In the presence of CT changes highly suggestive of COVID-19, with an initial negative RT-PCR, it is important to repeat a second swab within the next 24–48 hours to exclude a false-negative result, particularly common at the early stages and before symptoms onset. The RT-PCR swab sensitivity depends on the type of test used, the throat swab quality, and the viral load. Patients with more severe infections typically have higher viral loads in all locations and shed the virus longer (21 versus 14 days) [56].

Whenever possible, chest CT should be done in dedicated rooms or at times with fewer exams to avoid other patients and staff exposure [59].

The normal chest CT result is estimated to be about 10.6% (95% CI: 7.6%, 13.7%) in symptomatic COVID-19 patients [58]. Conversely, the normal chest CT incidence in asymptomatic COVID-19 patients is high and around 46% of patients [60].

In COVID-19 endemic areas, many studies suggest that an additional chest CT may be performed to help detect COVID-19 in patients who undergo extrathoracic CT [57]. These results can be observed in patients who underwent head and neck CT angiography [61–63], cervical or thoracic spine CT [61, 62], and abdomen CT [64–66]. At a later stage, a subsequent RT-PCR test to confirm COVID-19 diagnosis is required [67].

#### 5.2.2. Chest Radiography

Chest radiography is not sensitive for COVID-19 pneumonia due to the low ability to detect ground glass opacities, which are the main COVID-19 imaging features. Its role, therefore, should be restricted to follow-up in ICU patients, who are too fragile to be sent for CT [56], particularly with the concomitant immunosuppression.

#### 5.2.3. Chest Ultrasound

Several data indicate that lung US is a useful noninvasive bedside technique in the interstitial lung syndrome diagnosis and represents a reliable alternative to computed tomography (CT) during every step of COVID-19 disease, even before clinical manifestations [54, 68].

Unfortunately, chest US does not allow differentiation between bacterial pneumonia and viral pneumonia nor between pulmonary oedema and infection, and so its use should be limited to definitive-like cases only [69]. When US is unlikely to be diagnostic, CT should be preferred to minimize staff exposure. When instead US is recommended, for example, during pregnancy, in children or in ICU patients, it is a desirable bedside examination, reducing in this way the potential contamination of non-COVID areas during transport as well as the overall staff exposure [56].

Despite the wide availability, low cost, portability, and patient acceptability, particularly in those poorly cooperative, there are some limitations to an extensive US application in the setting of COVID-19, when compared to CT or X-ray [69]:

1. Increased time and closer exposure for the operators to patients and vice versa, in comparison to other diagnostic modalities; therefore, US has the potential to increase the chances of infection
2. Decontamination with liquid disinfectants is needed after COVID-19 examinations, and this might accumulate on the keyboard or in the command buttons
3. Digital instrumentation, such as the transducers of the screen, could be potentially damaged using an aggressive decontamination liquid, and at the same time, the effectiveness of standard cleaning tissues could not be sufficient; the correct disinfection modalities are still unknown to some operators
4. As for the case in the sterile theatre environment, the use of transparent, thin, disposable nylon bags might not entirely cover the US scanner but only the part in use at that given moment. Yet, this is not sufficient for the scanner to be utilized in non-COVID areas; thus, it is recommendable to provide each ward with a permanently located device, not to be shared with clean zones
5. There is no evidence regarding the reproducibility and univocal interpretation of lung US in the assessment of SARS-CoV-2 pulmonary involvement, and we know interoperator reproducibility is a major concern when it comes to US. There might be a smooth learning curve, although this is only supposed since it is not ethical to recruit personnel to be exposed to COVID-19 only for testing purposes

### 5.3. Follow-Up Post-LT

Patients should be followed up in their own centres when necessary, although we strongly encourage avoiding hospital visits for routine control visits and recommend online consultation via telemedicine. Probably, this will continue also later on, in the postpandemic scenario, especially for patients with reasonable established maintenance, where they could feel more comfortable in avoiding in-person visits with healthcare professionals [70].

However, remote consultation is not sufficient for patients with evidence of posttransplant complications, who therefore should attend in-person clinic and standard visits with the transplant staff. Symptoms such as fever, cough, and dyspnoea could be an early alarm for COVID-19; therefore, screening in accordance with the national/local protocol is recommended to intervene as early as possible [71].

Finally, LT recipients and candidates should be considered a high priority for vaccination strategies: surgery per se is associated with worse outcomes [2, 72], and often the underlying comorbidities of end-stage liver disease patients represent additional risk factors for higher mortality.

## 6. Conclusion

LT relies on an appropriate imaging method for its success; during the COVID-19 pandemic, it has been demonstrated that early signs of SARS-CoV-2 infection could be only detected by means of chest CT and that other screening modalities are less accurate. The increasing impact of radiology to limit the virus spread, joint to the early management of the disease complications, reinforces the need for a multidisciplinary approach in LT candidates and recipients; these frail patients benefit from close radiological monitoring. In consideration also of the necessity to reduce in-person visits and follow-up, it is possible that new routine exams will be performed aiming to early capture post-LT complications. This will also be applied to LT candidates or living donors to limit their hospital access but in this way provide a comprehensive view of the medical conditions and surgical anatomy.

## Figures and Tables

**Table 1:** Posttransplant checklist based on US findings.

Liver	Size and volume
	Focal lesions
	Hepatic echogenicity and ecostructure

Hepatic artery	Anatomy
	Patency and calibre
	Parietal calcifications
	Doppler wave in inspiration and after exhalation

Portal system	Anatomy
	Patency and calibre
	Doppler wave
	Varices
	Periportal oedema
	Intraportal gas

Biliary system	Anatomy
	Calibre
	Pneumobilia

Vascular/biliary anastomoses	Details of vascular/biliary anastomoses

Spleen	Size and volume
	Focal lesions
	Splenic artery
	Splenic vein

Lymph nodes	Periportal
	Portacaval

Other findings	Ascites
	Pleural effusion (right)

## Data Availability

The data supporting this review have been provided throughout the text.

## Abbreviations

CAP: Controlled attenuation parameter
COVID-19: Coronavirus Disease 2019
CT: Computed tomography
HCC: Hepatocarcinoma
ICU: Intensive care unit
LS: Liver stiffness
LT: Liver transplant
MRI: Magnetic resonance imaging
pSWE: Point shear wave elastography
RT-PCR: Reverse transcriptase-polymerase chain reaction
SARS-CoV-2: Severe Acute Respiratory Syndrome Coronavirus-type 2
SWE: Shear wave elastography
TE: Transient elastography
US: Ultrasound.
